# Sweet Cherry (*Prunus avium* L.) PaPIP1;4 Is a Functional Aquaporin Upregulated by Pre-Harvest Calcium Treatments that Prevent Cracking

**DOI:** 10.3390/ijms21083017

**Published:** 2020-04-24

**Authors:** Richard Breia, Andreia F. Mósca, Artur Conde, Sofia Correia, Carlos Conde, Henrique Noronha, Graça Soveral, Berta Gonçalves, Hernâni Gerós

**Affiliations:** 1 Centre of Molecular and Environmental Biology (CBMA), Department of Biology, Universidade do Minho, 4710-057 Braga, Portugal; richardgoncalvesbreia@gmail.com (R.B.); arturconde@bio.uminho.pt (A.C.); geros@bio.uminho.pt (H.G.); 2 Centre for the Research and Technology of Agro-Environmental and Biological Sciences (CITAB), Universidade de Trás-os-Montes e Alto Douro, 5001-801 Vila Real, Portugal; sofiacorreia@utad.pt (S.C.); bertag@utad.pt (B.G.); 3 Research Institute for Medicines (iMed.ULisboa), Faculty of Pharmacy, Universidade de Lisboa, 1649-003 Lisboa, Portugal; andreiafbm@medicina.ulisboa.pt (A.F.M.); gsoveral@ff.ulisboa.pt (G.S.); 4 Institute for Research and Innovation in Health (i3S), Universidade do Porto, 4200-135 Porto, Portugal; cconde@ibmc.up.pt; 5 Institute for Molecular and Cell Biology (IBMC), Universidade do Porto, 4200-135 Porto, Portugal; 6 Centre of Biological Engineering (CEB), Department of Engineering, Universidade do Minho, 4710-057 Braga, Portugal

**Keywords:** aquaporins, PaPIP1;4, calcium application, fruit-cracking, sweet-cherry

## Abstract

The involvement of aquaporins in rain-induced sweet cherry (*Prunus avium* L.) fruit cracking is an important research topic with potential agricultural applications. In the present study, we performed the functional characterization of PaPIP1;4, the most expressed aquaporin in sweet cherry fruit. Field experiments focused on the pre-harvest exogenous application to sweet cherry trees, cultivar Skeena, with a solution of 0.5% CaCl_2_, which is the most common treatment to prevent cracking. Results show that PaPIP1;4 was mostly expressed in the fruit peduncle, but its steady-state transcript levels were higher in fruits from CaCl_2_-treated plants than in controls. The transient expression of PaPIP1;4-GFP in tobacco epidermal cells and the overexpression of PaPIP1;4 in YSH1172 yeast mutation showed that PaPIP1;4 is a plasma membrane protein able to transport water and hydrogen peroxide. In this study, we characterized for the first time a plasma membrane sweet cherry aquaporin able to transport water and H_2_O_2_ that is upregulated by the pre-harvest exogenous application of CaCl_2_ supplements.

## 1. Introduction

Cracking induced by rain significantly limits production of sweet cherry fruits in regions of the world where rainfall occurs just before and during harvest [1]. Sweet cherry cracking is characterized by a strain-induced splitting of the outside layer as a consequence of an excessively positive water balance caused by water intake via both the fruit skin and the vascular system of the pedicel [2]. In rain-induced cherry cracking, microcracks concentrate water uptake to a given epidermal region [3,4], resulting in the bursting of individual flesh cells and the consequent leakage of cell contents into the apoplast [5,6]. The release of cell contents (especially malic acid) into the cell wall’s free space further weakens cell–cell adhesion, facilitating cracking propagation [4,7].

Studies on cherry cracking have focused on water movement at the plant and fruit levels [8,9,10], on the mechanisms involved in the deposition and cracking of the cuticle [11,12], and on the strain of the fruit skin [10,13,14,15]. In this regard, the possibility that increased water permeability could be mediated by aquaporins (AQPs) in more susceptible cultivars is a particularly attractive hypothesis. Transcriptomic data in *Litchi chinensis* suggests that several AQPs are differentially expressed in cracked fruits [16].

AQPs are transmembrane proteins that act as channels for the movement of water and/or small solutes through biological membranes [17]. Plasma membrane intrinsic proteins (PIPs) are the largest group of plant aquaporins [18,19]. Among the 35 full-length aquaporin genes in the *Arabidopsis* genome, 13 encode for PIPs. The PIP1 subgroup has five members (PIP1;1 to PIP1;5), whereas the PIP2 subgroup is represented by eight isoforms (PIP2;1 to PIP2;8) [20,21]. However, some aquaporins do not transport water or exhibit much broader specificity and are located in other intracellular membranes rather than in the plasma membrane. For instance, VvSIP1 (*Vitis vinifera* small basic intrinsic protein 1) and VvXIP1 (*V. vinifera* uncharacterized intrinsic protein 1)—which have been recently characterized by our group as water- and glycerol-transporting AQPs—are localized in the endoplasmic reticulum (ER) and in the ER/tonoplast, respectively [19,22].

Information about AQPs in sweet cherry and, in particular, how their expression/activity is influenced by currently experimented cherry cracking mitigation strategies remains scarce. Still, pivotal studies have contributed to boost the research in this exciting area, including the previous RNA-Seq analysis [23], the sequencing of the sweet cherry genome [24], and the recent identification of the sweet cherry aquaporin family (of which 16 members are expressed in the developing sweet cherry fruit, particularly in the flesh) [7].

Among cherry cracking mitigation, the exogenous application of biostimulants [25,26], glycine-betaine (GB) [27,28], abscisic acid (ABA) and methyljasmonate (MeJa) [29], and calcium formulations, which are the most common in the management of fruit cracking, have been tested [30,31]. In a recent study, the least cracking was observed for ABA + CaCl_2_- and GB + CaCl_2_-treated cherries, indicating an added benefit compared to spraying with CaCl_2_ alone [28].

As part of the ongoing project “CherryCrackLess” (PTDC/AGR-PRO/7028/2014), where the effect of the pre-harvest application of different compounds in combination with CaCl_2_ was tested, we aimed to functionally characterize cherry AQPs that could potentially be involved in fruit cracking. The present study targeted the cultivar Skeena, which is highly susceptible to cracking, and PaPIP1;4, because it is the most expressed aquaporin in cherry tissues, almost equally in the skin and flesh up to the harvest stage [7,23].

## 2. Results

### 2.1. PaPIP1;4 Sequence Analysis

**Figure 1:**
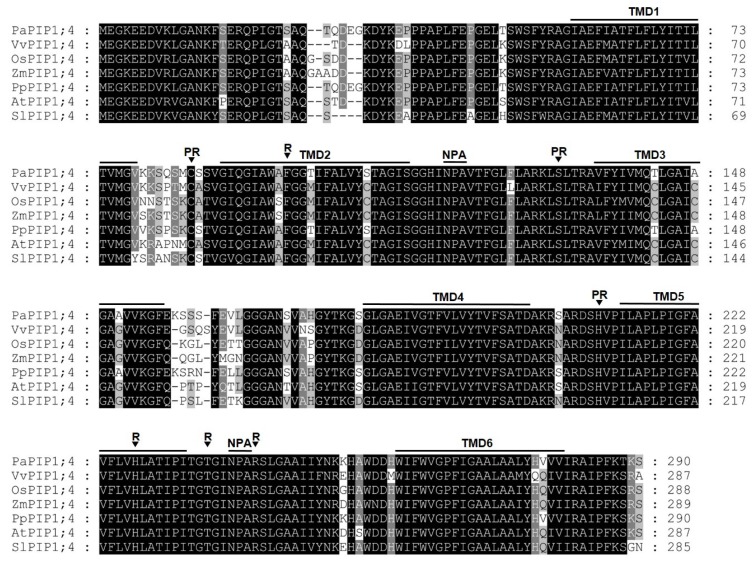
Alignment of PIP1;4 proteins showing six transmembrane domains (TMD1–TMD6), two intracellular and extracellular loops containing the conserved “NPA” motif, the four amino acids corresponding to the ar/R filter (R), and three conserved amino acid residues important for post-translational regulatory processes (PR).

The amino acid sequence of sweet cherry PIP1;4 was compared with its homologous proteins from peach (*Prunus persica*), grapevine (*Vitis vinifera*), rice (*Oryza sativa*), maize (*Zea mays*), thale cress (*Arabidopsis thaliana*), and tomato (*Solanum lycopersicum*) (Figure 1).

Remarkably, PIP1;4 amino acid sequences were highly conserved along the studied species. All proteins share six predicted transmembrane domains and the pore-forming loop with the two-signature motif Asn-Pro-Ala (NPA), which are a hallmark of aquaporins. Their ar/R filters (constituting the aromatic/arginine selectivity filter) consist of F/H/T/R residues, which are typical for the PIP subfamily that are most likely permeable to water [32]. Additionally, the Froger’s residues of the analyzed proteins are E/S/A/F/W, which are characteristic of an AQP but are not present in aquaglyceroporin [33]. Furthermore, PIP1;4 shares three conserved amino acid residues (C/S/H) that are potentially important for post-translational regulatory mechanisms [34].

### 2.2. Effect of Pre-Harvest Calcium Treatment in the Expression of PaPIP1;4 in Leaves, Fruits, and Peduncles of Sweet Cherry

**Figure 2:**
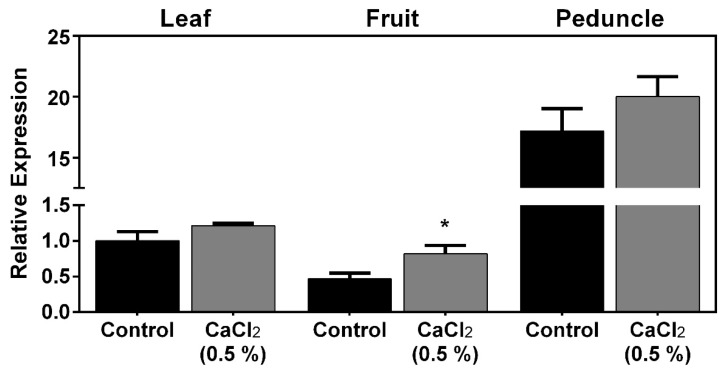
Steady-state gene expression of PaPIP1;4 in sweet cherry leaves, fruits, and peduncles treated with 0.5% CaCl_2_ or water (control). Values are the mean ± SD and asterisks indicate statistical significance (* *p* ≤ 0.05).

Results showed that PaPIP1;4 was more expressed in leaves than in fruits and was strongly expressed in the fruit peduncle (Figure 2).

As can be seen, the exogenous application of CaCl_2_ upregulated, by 2-fold, the expression of PaPIP1;4 in mature fruits. Further studies were then designed to functionally characterize PaPIP1;4 to test the hypothesis that PaPIP1;4 is a water-permeable aquaporin.

### 2.3. PaPIP1;4 Sub-Cellular Localization and Water Transport Capacity

**Figure 3:**
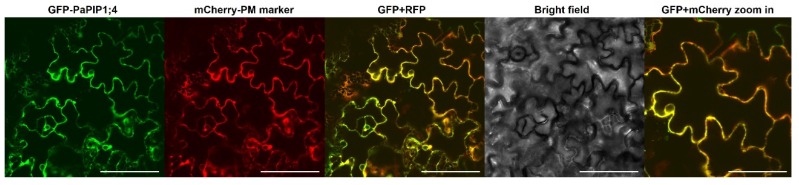
Subcellular localization of PaPIP1;4 in tobacco leaves. AtPIP2.1 was used as a plasma membrane marker [35]. Bar = 100 µm/200 µm in the zoom in box.

The sub-cellular localization of PaPIP1;4 was studied by the transient expression of the fusion GFP-PaPIP1;4 in *Nicotiana benthamiana* epidermal cells. The fusion AtPIP2.1-RFP was used as a plasma membrane marker [35]. Results showed that PaPIP1;4 co-localized with AtPIP2;1, indicating that PaPIP1;4 accumulates at the plasma membrane (Figure 3).

**Figure 4:**
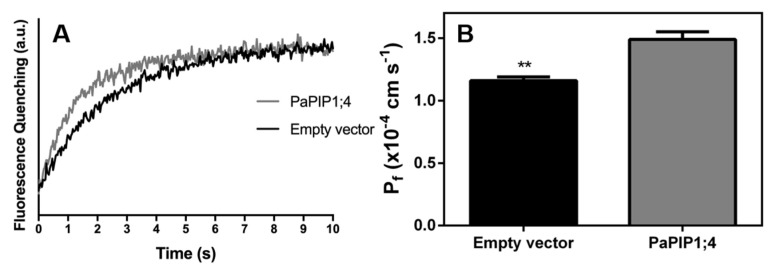
Water permeability of yeast cells transformed with pVV214-empty (control) or pVV214-PaPIP1;4 determined by stopped-flow fluorescence. (**A**) Fluorescence quenching of yeast cells subjected to a hyperosmotic shock and subsequent cell shrinkage; (**B**) Osmotic permeability coefficient (P*f*) of control and PaPIP1;4 transformed cells. Values are the mean ± SD and asterisks indicate statistical significance (** *p* ≤ 0.01).

To study the capacity of PaPIP1;4 to transport water across the plasma membrane, the yeast mutant YSH1172, deleted of endogenous aquaporins and impaired in water transport activity, was transformed with the pVV214-PaPIP1;4 construct, and its membrane water permeability was determined by stopped-flow spectrophotometry. The calculated osmotic water permeability coefficient (P*f*) was ca. 30% higher in PaPIP1;4 transformed yeast cells than in cells transformed with the empty vector (Figure 4).

Likewise, the activation energy (*E*_a_) for water transport was lower in PaPIP1;4 transformed yeast cells (14.4 kcal mol^−1^) than in control cells (15.4 kcal mol^−1^) (Table 1). Taken together, these results demonstrate that PaPIP1;4 can transport water across the plasma membrane.

**Figure 5:**
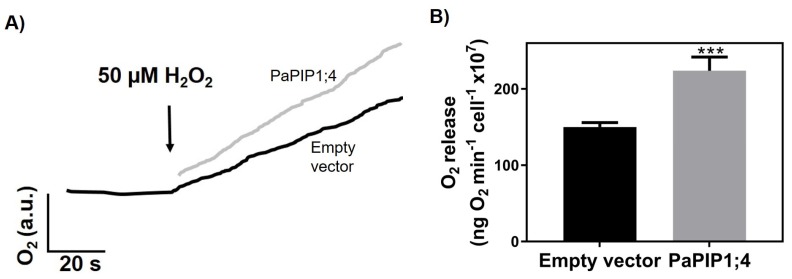
H_2_O_2_ transport by yeast cells transformed with pVV214-empty or pVV214-PaPIP1;4 (grey). (**A**) Typical O_2_ release (after intracellular breakdown of H_2_O_2_) signal recorded by the Clark electrode in response to 50 µM H_2_O_2_. (**B**) Rates of O_2_ release after the addition of 50 µM H_2_O_2_. Values are the mean ± SD and asterisks indicate statistical significance (*** *p* ≤ 0.001).

To study the capacity of PaPIP1;4 to transport H_2_O_2_, the rate of O_2_ release after the intracellular breakdown of H_2_O_2_ was studied with the Clark electrode in PaPIP1;4-expressing yeast cells (Figure 5).

Results showed that after the addition of 50 µM of H_2_O_2_, PaPIP1;4-expressing yeast cells released O_2_ at a higher rate (224 ± 18 ng O_2_ min^−1^ cell^−1^ × 10^7^) than control ones (150 ± 5.8 ng O_2_ min^−1^ cell^−1^ × 10^7^). The higher O_2_ release was due to an increased H_2_O_2_ uptake, which indicates that PaPIP1;4 is also able to transport this molecule.

## 3. Discussion

### 3.1. PaPIP1;4 Is an Aquaporin Upregulated by Pre-Harvest Application of CaCl_2_

The involvement of AQPs in cherry cracking may be explained, among other things, by their role in transcellular water movement from flesh to skin, or by allowing water partition between symplast and apoplast, alleviating both stress and strain on the skin of the growing fruit [7]. In this regard, considering the number of studies showing that CaCl_2_ application reduces rain-induced cracking [28,30,31], an overall decrease in the expression of aquaporins following this treatment could be expected. Paradoxically, we found that the expression of PaPIP1;4, which is the most expressed aquaporin in cherry tissues [7,23], increased following the application of 0.5% CaCl_2_. However, the occurrence of post-transcriptional regulatory mechanisms such as mRNA stability, protein biosynthesis, and subcellular trafficking and activity should not be ruled out, so that transport activity (see below) may not change in the same proportion as the steady-state transcript levels of PaPIP1;4 and other aquaporins expressed in the fruit.

The identified features of PaPIP1;4 point to an AQP that is able to transport water [33]. Interestingly, the PIP1;4 analyzed showed a difference in the first amino acid of the Froger’s residues (a Q or E) which has also been described in other species, namely, in strawberry [36]. Nonetheless, both are non-aromatic amino acids—a pre-requisite for water transport capacity [33]. Of the three conserved amino acids, the histidine residue could be involved in pH sensing [37], and the serine residue could be a possible target for phosphorylation, which crosstalks with Ca^2+^ [38]. Previous studies have already shown that aquaporin transport activity may be regulated by Ca^2+^. AQPs are phosphorylated in response to Ca^2+^ [39,40], and the direct effect of this cation on in vitro water transport activity is well documented, as in *Beta vulgaris* [41] and *Capsicum annum* [40]. In *Arabidopsis* under Ca^2+^ starvation, a transcriptomic analysis showed that the expression of several AQPs was modulated in the roots [42]. However, the hypothesis that the observed upregulation of PaPIP1;4 is mediated by Cl^−^ cannot be ruled out, and warrants future studies.

### 3.2. PaPIP1;4 Is Able to Mediate Water Transport in Yeast Cells

To the best of our knowledge, PaPIP1;4 is the first aquaporin to be functionally characterized in sweet cherry, although the P*f* value only increased by ca. 30% relative to the control. This could result from a low level of expression in the yeast model used, but could also be due to the low water transport activity generally associated with PIP1 members [43]. Reports showing that PIP1s are bona fide water transporters [44,45] are in agreement with our results, but PIP1s may also be involved in the regulation of PIP2 activity by forming heterotetramers [46]. Thus, the co-expression of PIP1s with PIP2s increases water transport activity [47]. In this regard, the co-expression of PaPIP1;4 and PaPIP2s putatively regulated by CaCl_2_ exogenous application could bring important insights into the function of PaPIP1;4 in response to pre-harvest treatments with calcium-based formulations.

In summary, our results confirm the hypothesis that PaPIP1;4, previously identified as one of the most expressed aquaporins in cherry tissues [7,23], is indeed a bona fide water transporter, and showed that PaPIP1;4 expression was upregulated in response to pre-harvest CaCl_2_ application. Nonetheless, other aquaporins, like the ones recently identified in sweet cherry fruit during development and ripening [7], may play pivotal roles in transmembrane water transport. Thus, the functional characterization of these proteins, and the study of their relative expression in sensitive and resistant cultivars and their response to pre-harvest cracking mitigation treatments, will provide import insight to clarify whether or not aquaporins are key players in sweet cherry cracking.

## 4. Materials and Methods

### 4.1. Plant Material and Treatment

Field experiments were performed in an orchard located in Carrazedo de Montenegro, Portugal (latitude 41°33’N, longitude 7°17´W, altitude 682 m). In this study, we used six-year-old sweet cherry trees, cv. Skeena, grafted on “Gisela 6” rootstock. Trees were spaced 4.5 m between rows and 2.0 m from each other in the row trained under a vertical system. Nine trees were selected per treatment, which focused on the application of water (control) or a solution with 0.5% CaCl_2_. All applications were mixed with a wetting agent (0.1%). Treatments were applied 56, 62, and 69 days after full bloom (DAFB). Fruits, peduncles, and leaves from each tree were harvested simultaneously at the commercial maturity stage and frozen in liquid nitrogen.

### 4.2. In Silico Studies

The PIP1;4 sequence was obtained from the database of the National Center of Biotechnology (NCBI). Protein alignment was performed by Prankster, and the result was visualized in Genedoc [48]. Topology predictions were performed with TOPCONS [49].

### 4.3. Sequence Accession Numbers

PaPIP1;4 (XP_021833015.1), PpPIP1;4 (XP_007218768.1), AtPIP1;4 (NP_567178.1), VvPIP1;4 (ABH09324.1), OsPIP1;4 (XP_015635466.1), ZmPIP1;4 (ACF84511.1), SlPIP1;4 (NP_001234413.2).

### 4.4. RNA Isolation from Leaves, Fruits, and Peduncles

An initial amount of 200 mg of ground sample was used for total RNA extraction, following the method described by [50] combined with the GRS Total RNA kit-Plant (GRISP). cDNA was synthesized from 1 µg of total RNA using the Xpert cDNA Synthesis Master-mix Kit (GRISP).

### 4.5. Real-Time PCR Studies

Quantitative real-time PCRs were prepared with Xpert Fast SYBR Blue (GRISP) using 1 µL of diluted cDNA (1:10) in 10 µL of reaction mixture per well. For reference genes, PaACT1 (actin) was used [51]. The specific primer pairs used for each studied gene are listed in the Table 1. Normalization of the average expression value of the reference gene was performed as described by [52].

### 4.6. PaPIP1;4 Molecular Cloning and Construction of Destination Vectors

PaPIP1;4 was cloned using Gateway^®^ technology. Primers bearing the *attB* sequences (Table 1) for site-specific recombination with the entry plasmid pDNOR221 were used for PCR amplification of the target genes. Subsequently, recombination of the target genes containing the *attB* sites with the entry plasmid was performed by the BP clonase enzyme. The target genes carried in the entry plasmid were then recombined by the LR clonase enzyme into the pH7WGF2 plasmid (containing the *egfp* gene) for sub-cellular localization and into the pVV214 plasmid for heterologous expression in *Saccharomyces cerevisiae*. All constructs were confirmed by sequencing.

### 4.7. Subcellular Localization of PaPIP1;4

The pH7WGF2-GFP-PaPIP1;4 construct was introduced in *Agrobacterium tumefaciens* (EHA105 strain), and transient transformation of tobacco (*Nicotiana benthamiana*) leaf epidermal cells was performed according to [53]. Transformed *Agrobacterium* cells were cultivated overnight in liquid LB medium up to the exponential–stationary phase and then diluted to OD_600 nm_ = 0.1 with infiltration buffer (50 mM MES pH 5.6, 2 mM Na_3_PO_4_, 0.5% glucose, and 100 μM acetosyringone). Diluted cells were cultivated again until the culture reached an OD_600 nm_ of 0.2. Four-week-old tobacco plants were infiltrated with the bacterial cultures and leaf discs were examined under the confocal microscope after 3 days. The used plasma membrane marker was the AtPIP2.1 [35]. The marker was constructed in an mCherry harboring plasmid and co-expressed with pH7WGF2-GFP-PaPIP1;4.

### 4.8. Functional Characterization of PaPIP1;4 by Stopped-Flow Fluorescence

The *Saccharomyces cerevisiae* strain YSH1172 (MATα *leu2::hisG trp1::hisG his3::hisG ura3-52 aqy1::KanMX4 aqy2::HIS3*) [54], which is a deletion mutant for aquaporins, was transformed with pVV214-PaPIP1;4 and with pVV214 (control) constructs to functionally characterize PaPIP1;4. The stopped-flow technique was used to monitor cell volume changes induced by osmotic shocks in cells loaded with a concentration-dependent, self-quenching fluorophore [55]. Cells were pre-loaded for 10 min at 30 °C with the nonfluorescent precursor 5-and-6-carboxyfluorescein diacetate (CFDA, 1 mM in isosmotic solution), which is cleaved by intracellular nonspecific esterases generating the fluorescent form, and expected to remain mainly in the cytoplasm. Although some of the probe may be either accumulated in the vacuole or exported to the medium [56], this effect can be neglected in light of the very rapid water flow. As the cells shrink in response to osmotic changes, the concentration of the entrapped fluorophore increases with a corresponding change in the fluorescence output [57]. To avoid pH interference in fluorescence during the osmotic shock, cell suspensions and osmotic solutions were buffered with K^+^-citrate/KH_2_PO_4_ 50 mM at the selected pH. Experiments were performed on a HI-TECH Scientific PQ/SF-53 stopped-flow apparatus, which has a 2 ms dead time, controlled temperature, interfaced with an IBM PC/AT compatible 80,386 microcomputer. Four runs were usually stored and analyzed in each experimental condition. In each run, 0.1 mL of cell suspension (initial osmolarity (osm_out_)_o_ = 1.4 M) was mixed with an equal amount of hyperosmotic sorbitol solution (final tonicity = 1.5) to reach an inward-directed solute gradient and induce an outward water flow responsible for cell volume change. Fluorescence was excited using a 470 nm interference filter and detected using a 530 nm cut-off filter. The time course of volume change was followed by fluorescence quenching CFDA. The recorded fluorescence signals were fitted to a single exponential, from which the rate constant (k) was calculated. The osmotic water permeability coefficient (P*f*) was estimated from the linear relationship between P*f* and k, P*f* = k(V_o_/A)(1/V_w_(osm_out_)_∞_), where V_w_ is the molar volume of water, V_o_/A is the initial volume to area ratio of the cell population, and (osm_out_)_∞_ is the final medium osmolarity after the osmotic shock. The activation energy of water transport was determined by stopped-flow experiments performed at temperatures ranging from 9 to 37 °C. The activation energy (*E*_a_) of water transport was evaluated from the slope of Arrhenius plots (lnP*f* as a function of 1/T).

### 4.9. Clark Electrode Assays

The YSH1172 *aqy*-null *Saccharomyces cerevisiae* strain was transformed with pVV214-PaPIP1;4 and pVV214-empty (control) constructs and pre-cultured in YNB+SC solid medium. Liquid cultures were then grown overnight. Cells were washed three times and resuspended in water to a final OD_600 nm_ = 1.0. H_2_O_2_ was added to the cell suspension to a final concentration of 50 μM, and the O_2_ formation was followed with a Clark electrode coupled to an YSI 5300 Biological Oxygen Monitor as described previously [22].

### 4.10. Statistical Analysis

Results were statistically analyzed by Student’s *t*-test and by analysis of variance test (one-way) using Prism v. 5 (GraphPad Software, Inc.). Post-hoc multiple comparisons were performed using Tukey’s HSD test. For each condition, differences between mean values are identified with different letters or asterisks.

## Figures and Tables

**Table 1:** Permeability coefficient (P*f*) and activation energy (*E*_a_) for water transport in yeast membranes obtained by stopped-flow spectroscopy.

	P*f* (10^−4^ cm s^−1^)	*E*_a_ (kcal mol^−1^)
Empty vector	1.16 ± 0.03	15.4 ± 0.034
PaPIP1;4	1.49 ± 0.06 *	14.4 ± 0.038 *

Asterisk denotes statistical significance (* *p* ≤ 0.05).
